# Communication and Cooperation Between the Medical Academy, Medical Association, and Local Government: Health Counseling Program After Recovery From Coronavirus Disease 2019 (COVID-19) in Daegu

**DOI:** 10.3389/fpubh.2020.563757

**Published:** 2021-01-13

**Authors:** Yun-A Kim, Geon Ho Lee, Keun-Mi Lee, Hae-Jin Ko, DongWook Lee, A-Sol Kim

**Affiliations:** ^1^Department of Family Medicine, Daegu Catholic University School of Medicine, Daegu, South Korea; ^2^Department of Family Medicine, Yeungnam University Medical Center, Yeungnam University College of Medicine, Daegu, South Korea; ^3^Department of Family Medicine, School of Medicine, Kyungpook National University, Daegu, South Korea; ^4^Department of Family Medicine, School of Medicine, Dongguk University, Gyeongju, South Korea

**Keywords:** COVID-19, medical academia, medical association, local government, consultation

## Abstract

We are currently experiencing the disaster of the COVID-19 pandemic. Since the first case of Coronavirus disease 2019 (COVID-19) was confirmed in South Korea on January 20, the number of COVID-19 cases in South Korea has been rapidly increasing until early March due to a local spread in Daegu, which is one of the eight metropolitan cities in South Korea with a population of 2.5 million. As the medical academy has social accountability as professionals, Daegu-Gyeongbuk branch of the Korean Academy of Family Medicine (Daegu-Gyeongbuk branch) developed the health counseling program for discharged COVID-19 patients. The Daegu-Gyeongbuk branch communicated with Daegu Medical Association and Daegu city for this program and incorporated available resources and capabilities as a leader of this program. This newly developed counseling program consists of medical consultations, sending healthcare brochures and medical supplies, and the appraisal at the end of the program. Not only COVID-19 related symptoms but also other psychological problems are also dealt with during consultations. This program started on March 18, and over 1,700 recovered patients have been receiving counseling as of April 28. Communication and cooperation between the medical academy, medical association, and government are essential to overcome the COVID-19 pandemic. Besides, we expect to apply this health counseling program and our model of setting this program cooperating with medical association and government to different infectious pandemic crisis.

## Introduction

Twenty-seven cases of pneumonia of unknown origin were reported by the China National Health Commission on December 31, 2019 in Wuhan, Hubei province (1). This unidentified pneumonia was later revealed to be due to a new coronavirus (2019-nCov) (2), which has been spreading rapidly, reaching 1,210,956 confirmed cases and 67,594 deaths worldwide as of April 6, 2020 (3). Since the first case of 2019-nCov disease, named Coronavirus disease 2019 (COVID-19) was confirmed in South Korea on January 20 (4), the 31st case of COVID-19 was confirmed in Daegu, which is one of the eight metropolitan cities in South Korea with a population of 2.5 million as first local case on February 18 (5). Since then, the number of COVID-19 cases in Daegu has been rapidly increasing until early March mostly related to religious events. As of March 9, the total number of confirmed cases in South Korea was 7,382 and 5,571 in Daegu which accounts for about 67% of overall cases in South Korea (6). Considering this rapid increase of confirmed cases in Daegu and the possible assumption that many patients will be discharged accordingly, an emergency board meeting of the Daegu-Gyeongbuk branch of the Korean Academy of Family Medicine (Daegu-Gyeongbuk branch) was held to determine how to cope with COVID-19, on March 9 and 17th. Among several suggestions during the meeting, the publication of healthcare brochures and the health counseling program for discharged COVID-19 patients were accepted. In this perspective, we aimed to introduce how we cooperated with medical association and local government to set this health counseling program in COVID-19 pandemic. This study was approved by the institutional review board of Daegu Catholic University Medical Center (IRB approval number: CR-20-164-L).

## Cooperation Between The Medical Academy, Medical Association, and Local Government

The health counseling program after recovery from COVID-19 is a newly developed medical counseling program by family physicians at the Daegu-Gyeongbuk branch based on phone consultations. The purposes of the health counseling program focus on the management of medical and psychological problems and early recognition of coronavirus reactivation. Thus, not only medical symptoms associated with COVID-19 but also other psychological problems such as depression, anxiety, and even family relations are included in the counseling process. In addition, physicians provide emotional support to individuals in every counseling session. This program was scheduled to be held for a month for each participant.

**Figure 1:**
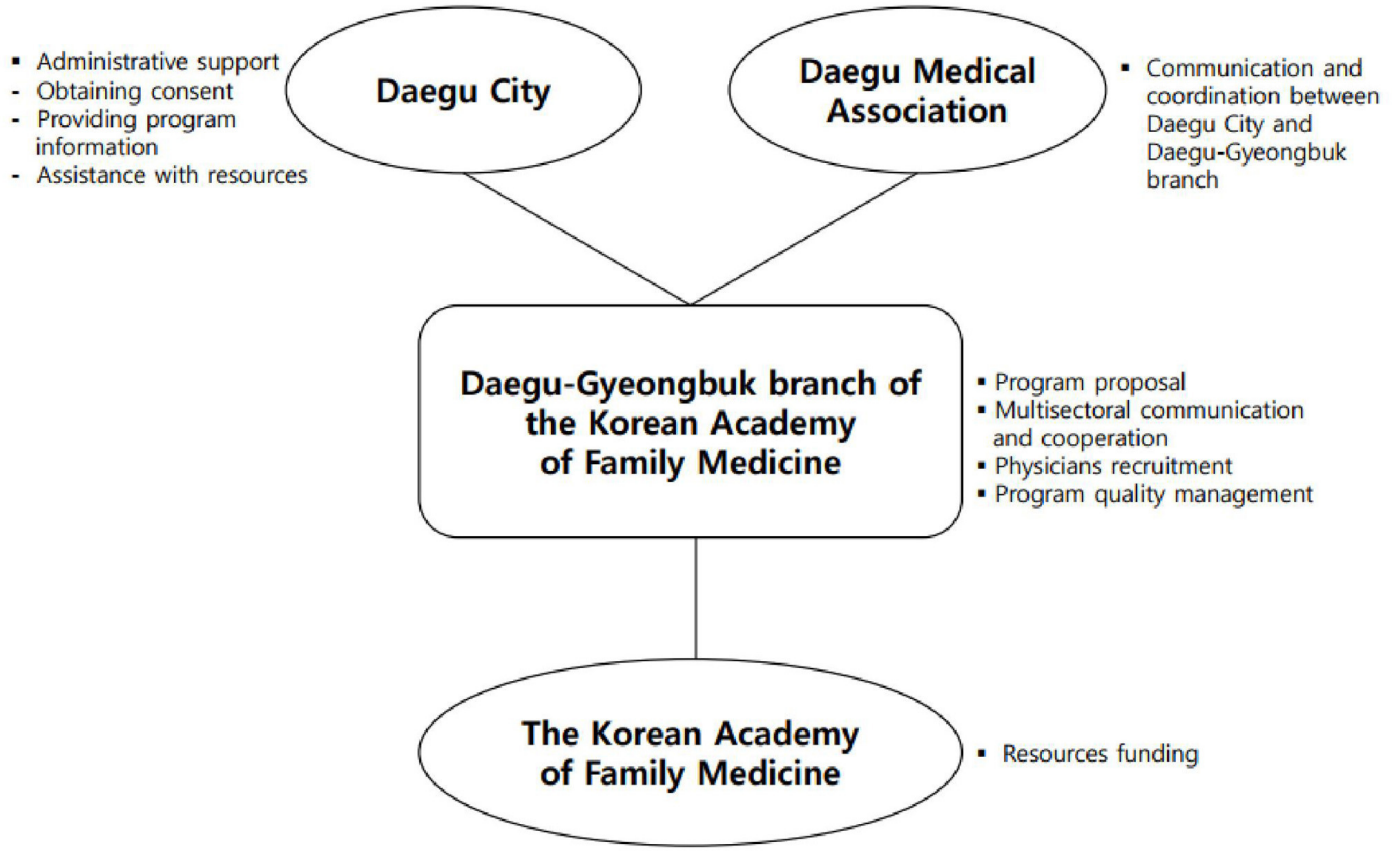
Physician-based intervention model in cooperation with the government and medical association.

As the first step to start this program, the Daegu-Gyeongbuk branch proposed this program to the Daegu Medical Association and Daegu city. In the emergent situation of rapid spread of COVID-19, the Daegu-Gyeongbuk branch communicated and cooperated closely with Daegu city and Daegu Medical Association reaching an agreement that management for patients who recovered from COVID-19 is necessary. As a rapid response to the proposal of this program, Daegu city was responsible for introducing this newly developed program to discharged Daegu citizens, getting the consent for program participation, and providing individual information including the date of isolation and discharge to the Daegu-Gyeongbuk branch. Daegu city also provides resource assistance, such as mobile phones for consultations. Daegu Medical Association has helped in terms of communication and coordination between Daegu city and the Daegu-Gyeongbuk branch. Furthermore, the Korean Academy of Family Medicine provided donations to the Daegu-Gyeongbuk branch. As a leader of this program, the Daegu-Gyeongbuk branch contributed by coordinating each organization's role as well as suggesting this program. Besides, the Daegu-Gyeongbuk branch distributed funding resources to program participants based on the principles of justice. Regarding physicians, the Daegu-Gyeongbuk branch recruited volunteers to counsel the recovered COVID-19 patients, and a total of 20 family physicians decided to participate in the program. All these processes are briefly demonstrated in Figure 1. Finally, this program started on March 18 for COVID-19 patients who recovered and resided in Daegu city.

**Figure 2:**
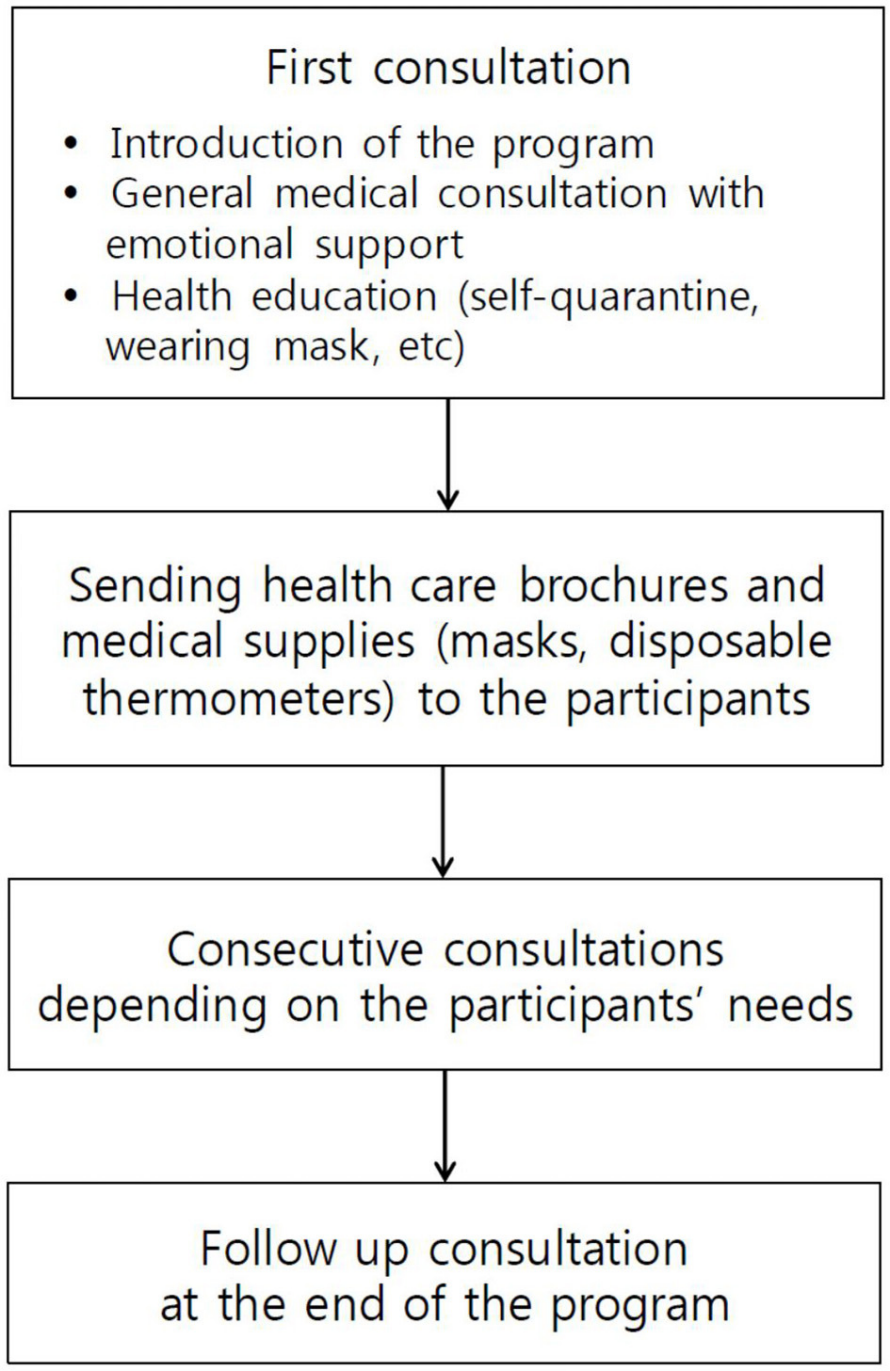
Flowchart of the health counseling program after recovery from the Coronavirus disease 2019 (COVID-19).

As shown in Figure 2, the program consists of two regular consultations at the beginning and the end of the program. During the first consultation, physicians introduce the program and obtain baseline information such as underlying diseases, first symptoms of COVID-19, and current symptoms. Sleep, mood, stress, and family relations are also dealt with during consultations. According to a counselee's condition, physicians provide proper medical recommendations and emotional support individually, and consecutive consultations might be conducted based on the participant's needs. Healthcare brochures and medical supplies such as a set of masks and disposable thermometers are also sent to the participants with funding resources from the Korean Academy of Family Medicine mentioned above. In addition, a mobile questionnaire survey regarding sleep, depression, anxiety, stress, quality of life, and family relations has also been planned during this program under the Daegu-Gyeongbuk branch's supervision.

## Overall Finding

A total of 1,706 recovered patients have been receiving counseling, which means 25% of all recovered patients from COVID-19 in Daegu have had medical consultation. Most of the participants were female (66.2%), and the mean age was 39.6 years (range: 0–79 years). This program ended at the 1st week of June considering new confirmed cases in Daegu have declined since mid-April. During this program, we assessed recovered patients' depression, anxiety, stress, quality of life, and family relations along with clinical presentations of COVID-19.

Here is an example of actual consultation; A 26-year-old woman who was confirmed as COVID-19 on March 4 and released from quarantine on March 19 had first consultation on March 24. She has had asthma and still complained of mild dyspnea in the first consultation. She also complained of mild anxiety and depression from stigma attached with COVID-19. Her father and her colleagues treated her as an infection source even after discharge with twice negative on COVID-19 tests. Counseling physician recommended watchful waiting for mild dyspnea since she did not have other related symptoms and its course was getting better per participant. However, she seemed to need further consultation for anxiety and depression. So, the physician recommended follow up consultation a week later, and introduced COVID-19 mental health hot line which is available for 24 h a day. On the 2nd consultation, the participant said she felt much better for her breathing, but still anxious to meet other people. Though, she mentioned that she felt somewhat relieved after having conservation with the physician. In the final consultation, the participant said she went back to work without any symptoms, but she was still cautious when talking with other people even with her mask on. The physician ended the counseling program congratulating her recovery from COVID-19, and emphasizing the health education again such as hand hygiene and wearing mask when going for work.

According to the participant satisfaction survey at the end of the program, about 78% of participants were satisfied with the consultation, 16% reported neither positive nor negative, and only 6% were dissatisfied with the consultation. As reasons for satisfaction with this consultation, health education including hygiene education to prevent COVID-19 and early recognition against COVID-19, and emotional support such as active listening and showing empathy was mainly selected. On the other hand, a lack of empathy was presented as a reason for dissatisfaction. According to the physician satisfaction survey, 7 physicians among 20 reported their routine work was disturbed a little. However, most of the physicians reported they felt overwhelmed and proud to have been part of this newly developed program as medical professionals in the COVID-19 pandemic. One of limitations that physicians pointed was immediate testing or prescribing was impossible due to phone-based consultation.

## Discussion

We are currently experiencing the disaster of the COVID-19 pandemic. Rapid diagnosis, treatment, and isolation of confirmed or suspected cases are essential to cope with this global health crisis. Along with these actions, the management strategy for patients who have recovered from COVID-19 is also crucial. To the best of our knowledge, this is the first health counseling program for patients who have recovered from the COVID-19 pandemic, conducted by physicians. This phone-based counseling program surveyed not only current remain symptoms which might be the early signs of coronavirus reactivation, but also each participants' mental health. However, there are some limitations in this program. First, this health counseling program might not be well-organized since it was urgently designed, and promptly performed responding to COVID-19 outbreak in Daegu. The performance evaluation for both physicians and participants has been completed and being analyzed in regard to planning, action, and overall satisfaction. Second, we could not involve all patients recovered from COVID-19, and this program was limited to only Daegu citizens. Thus, individualized adaptation should be required when this program is applied to different government or country. Last, we could not evaluate this program in terms of comparison with other counseling programs since the organized counseling program by physician in COVID-19 pandemic has not been reported yet. Considering that COVID-19 is still ongoing and over hundreds of thousands of patients will be discharged worldwide, communication and cooperation between the medical academy, medical association, and government are essential to overcome the COVID-19 pandemic. Moreover, as the medical academy and medical association have social accountability as professionals, they should provide leadership and cooperation between national authorities and other medical associations to incorporate available resources and capabilities during this pandemic crisis. Besides, we expect to apply this health counseling program and our model of setting this program cooperating with medical association and government to different infectious pandemic crisis.

## Data Availability Statement

The raw data supporting the conclusions of this article will be made available by the authors, without undue reservation.

## Ethics Statement

The studies involving human participants were reviewed and approved by the institutional review board of Daegu Catholic University Medical Center. The patients/participants provided their written informed consent to participate in this study.

## Author Contributions

GHL developed the research questions, provided critical revision of the article, and provided final approval of the version to publish. KML, HJK, DWL, and ASK provided substantial contributions to the program management and supervision. YAK contributed to writing and submitting the manuscript. All authors contributed to the article and approved the submitted version.

## Conflict of Interest

The authors declare that the research was conducted in the absence of any commercial or financial relationships that could be construed as a potential conflict of interest.
